# Antitumor effects of duvelisib on Epstein–Barr virus‐associated lymphoma cells

**DOI:** 10.1002/cam4.1311

**Published:** 2018-03-09

**Authors:** Jun‐ichi Kawada, Shotaro Ando, Yuka Torii, Takahiro Watanabe, Yoshitaka Sato, Yoshinori Ito, Hiroshi Kimura

**Affiliations:** ^1^ Departments of Pediatrics Nagoya University Graduate School of Medicine 65 Tsurumai‐cho, Showa‐ku Nagoya 466‐8550 Japan; ^2^ Departments of Virology Nagoya University Graduate School of Medicine 65 Tsurumai‐cho, Showa‐ku Nagoya 466‐8550 Japan

**Keywords:** Duvelisib, EBV, lymphoma, PI3k/Akt, PI3K*δ*

## Abstract

Epstein–Barr virus (EBV) is a ubiquitous oncogenic virus that is associated with B cell lymphomas, including Burkitt lymphoma and Hodgkin lymphoma. Previous studies have shown that the phosphatidylinositol 3‐kinase (PI3K)/Akt pathway is activated in EBV‐associated lymphomas and can be a novel therapeutic target. An oral dual inhibitor of PI3K*γ* and PI3K*δ*, duvelisib, is in clinical trials for the treatment of lymphoid malignancies. In this study, we evaluated how duvelisib affects the activity of the PI3K/Akt signaling pathway and if it has antitumor effects in EBV‐associated lymphoma cell lines. We found that the PI3K/Akt signaling pathway was activated in most of the B and T cell lymphoma cell lines tested. Additionally, duvelisib treatment inhibited cellular growth in the tested cell lines. Overall, B cell lines were more susceptible to duvelisib than T and NK cell lines in vitro regardless of EBV infection. However, the additional influence of duvelisib on the tumor microenvironment was not assessed. Duvelisib treatment induced both apoptosis and cell cycle arrest in EBV‐positive and ‐negative B cell lines, but not in T cell lines. Furthermore, duvelisib treatment reduced the expression of EBV lytic genes (BZLF1 and gp350/220) in EBV‐positive B cell lines, suggesting that duvelisib suppresses the lytic cycle of EBV induced by B cell receptor signaling. However, duvelisib did not induce a remarkable change in the expression of EBV latent genes. These results may indicate that there is therapeutic potential for duvelisib administration in the treatment of EBV‐associated B cell lymphomas and other B cell malignancies.

## Introduction

Epstein–Barr virus (EBV) is the primary agent causing infectious mononucleosis and persists asymptomatically for life in nearly all adults. EBV infection is associated with the development of B cell lymphomas, posttransplant lymphoproliferative disease (PTLD), natural killer (NK)/T cell lymphoma, and chronic active EBV disease 1, 2, 3, 4. Generally, EBV‐PTLD disease results in a type III latency pattern characterized by the expression of all EBV latency‐associated proteins. These include the EBV nuclear antigens (EBNA) 1, 2, 3A, 3B, and 3C (EBNA‐1, EBNA‐2, EBNA‐3A, ‐3B, and ‐3C, respectively) and latent membrane proteins (LMP) 1 and 2 (LMP1, LMP2, respectively). Conversely, EBV‐positive Burkitt lymphoma is usually characterized by a type I latency pattern and primarily expresses EBNA‐1 1. Patients with EBV‐associated lymphoid malignancies treated with cytotoxic chemotherapy are often refractory to it. Therefore, understanding the underlying molecular pathways of EBV‐associated lymphoid malignancies is necessary to develop more effective treatment strategies.

LMP1 is considered a major EBV oncoprotein that mediates activation of multiple cellular signaling pathways, such as c‐Jun‐N terminal kinases (JNK), tumor necrosis factor (TNF) receptor associated factor TRAF, and nuclear factor kappa beta (NF‐*κ*B) signaling 5, 6. In 2003, Dawson et al. demonstrated that LMP1 can also activate phosphatidylinositol 3‐kinase (PI3K) and induce subsequent activation of serine/threonine protein kinase (Akt) to promote cell survival 7. Subsequently, several other studies have shown that constitutive activation of the PI3K/Akt pathway via LMP1 is a key element of LMP1‐mediated transformation. This suggests that the PI3K/Akt pathway can be a therapeutic target for the treatment of EBV‐associated lymphoid malignancies 8, 9, 10, 11. Furthermore, a recent study using genome‐wide CRISPR/Cas9 screens in EBV‐transformed B cells has revealed that the EBV oncoprotein LMP2A also activates the PI3K/Akt pathway 12.

The PI3K/Akt signaling pathway has been associated with many virus‐associated cancers. Moreover, it is known to regulate numerous biological activities, including cellular growth, survival, and proliferation 13, 14. The PI3Ks are divided into three classes: I, II, and III. Of the three classes of PI3K isoforms, class I PI3K comprises four different catalytic isoforms including PI3K*α*,* β*,* γ*, and *δ* 15. While PI3K*α* and *β* are ubiquitously expressed in all mammalian tissues, PI3K*γ* and PI3K*δ* have more selective roles. PI3K*γ* has a particular role in T cell activation and PI3K*δ* expression is largely restricted to hematopoietic cells, and has a crucial role in mediating B‐cell receptor (BCR) signaling, proliferation/survival, and migration 16. Furthermore, PI3K*δ* is aberrantly activated in a variety of B cell malignancies such as chronic lymphocytic leukemia (CLL) and acute myeloid leukemia 17, 18. Therefore, PI3K*δ* has emerged as a promising therapeutic target in hematological malignancies. In fact, in a phase III clinical trial testing the oral selective PI3K*δ* inhibitor idelalisib in combination with rituximab, the survival of CLL patients improved considerably 19.

Duvelisib is a molecule previously found to inhibit the PI3K/Akt signaling pathway, thereby inhibiting BCR signaling, diminishing chemotaxis, and inhibiting cytokine‐induced CLL cell proliferation with minimal apoptosis 20, 21. The duvelisib molecule closely resembles the chemical structure of idelalisib, but biochemically targets PI3K*γ* in addition to PI3K*δ* 22. It is expected that dual inhibition of PI3K*γ* and PI3K*δ* by duvelisib may be another therapeutic target for the treatment of CLL and may overcome resistance formed against idelalisib 23. Furthermore, clinical studies of duvelisib in indolent non‐Hodgkin lymphoma and CLL have shown clinical activity 20, 24. However, the effects of PI3K*γ* or PI3K*δ* inhibitors on EBV‐associated lymphoma cells have not been investigated. In this study, we evaluated the activity of the PI3K/Akt signaling pathway and antitumor effects of duvelisib on EBV‐associated lymphoma cell lines.

## Materials and Methods

### Cell lines and reagents

The cell lines used in this study are summarized in Table 1. Lymphoblastoid cell line (LCL) was generated by infection of B cells with EBV (B95‐8 strain). Akata (+) 25, Mutu I 26, Raji 27, and P3HR1 28 are EBV‐positive B cell lines, and BJAB 29 and Akata (‐) 30 are EBV‐negative B cell lines. SNT16 31 is an EBV‐positive T cell line, and Jurkat 32 and MOLT4 33 are EBV‐negative T cell lines. KAI3 34 is an EBV‐positive, and KHYG1 35 is an EBV‐negative NK cell line. Duvelisib was obtained from Infinity Pharmaceuticals (Cambridge, MA) and was dissolved in DMSO. Idelalisib was purchased from Tokyo Chemical Industry (Tokyo, Japan) and was dissolved in DMSO.

**Table 1:** Characteristics of cell lines

Cell type	Cell line	EBV (latency pattern)	Cell origin
B cell lines	BJAB	‐	Burkitt lymphoma
	Akata (‐)	‐	EBV‐negative clones from Akata
	Akata (+)	+ (I)	EBV‐related Burkitt lymphoma
	Mutu I	+ (I)	EBV‐related Burkitt lymphoma
	P3HR1	+ (II)	EBV‐related Burkitt lymphoma
	LCL	+ (III)	Primary B cells transformed with EBV
	Raji	+ (III)	EBV‐related Burkitt lymphoma
T cell lines	Jurkat	‐	Acute T lymphoblastic leukemia
	MOLT4	‐	Acute T lymphoblastic leukemia
	SNT16	+ (II)	Chronic active EBV disease
NK cell lines	KHYG1	‐	Aggressive NK cell leukemia
	KAI3	+ (II)	Chronic active EBV disease

LCL, lymphoblastoid cell line.

### Cell proliferation

Cells (2 × 10^5^/mL) were cultured for 48 and 72 h in the presence of duvelisib or idelalisib at concentrations from 0.1 to 5 *μ*mol/L. Viable cells were determined using a Countess^™^ Automated Cell counter (Invitrogen, Carlsbad, CA). Subsequent experiments were performed in triplicate at minimum.

### Detection of PI3K/Akt signaling by immunoblotting

Collected cell extracts were diluted in sample buffer, and prepared for SDS‐PAGE. Equal amounts of cellular protein extracts were loaded on 4–15% polyacrylamide gel. *β*‐actin was used as a loading control. The separated protein bands were transferred to PVDF membranes, prior to antibody incubation. Membranes were incubated with primary antibodies at 4°C with gentle shaking, overnight. The primary antibodies were as follows: Akt, phospho‐Akt (Thr308), PI3K*γ*, PI3K*δ*, and caspase‐3 (Cell Signaling Technology, Beverly, MA), poly ADP ribose polymerase (PARP) and *β*‐actin (Sigma‐Aldrich, St. Louis, MO).

### Apoptosis detection with Annexin V by flow cytometry

Apoptosis was assessed using an Annexin V‐PE/7‐AAD Apoptosis Detection Kit (BD Pharmingen Biosciences, San Diego, CA), following manufacturer's instructions. Briefly, cells (2 × 10^5^/mL) were seeded in 24‐well plates and treated with 5 *μ*mol/L of duvelisib or DMSO for 48 h. Cells were then washed in PBS and incubated with Annexin V‐phycoerythrin (PE) in a buffer containing 7‐aminoactinomycin D (7‐AAD). Apoptotic cells (7‐AAD negative, PE Annexin V positive) were analyzed using flow cytometry.

### Cell cycle analysis

Cells (2 × 10^5^/mL) were treated with 5 *μ*mol/L of duvelisib or DMSO for 48 h. Cells were then fixed with ice‐cold 70% ethanol, and then washed with PBS. Fixed cells were re‐suspended in 50 *μ*g/mL propidium iodide (PI) solution (Sigma‐Aldrich) with DNase‐free RNase, and then analyzed using flow cytometry. Experiments were performed in triplicate.

### Real‐time RT‐PCR

RNA was extracted from 1 × 10^6^ cells from the culture medium with the QIAamp RNeasy Mini Kit (Qiagen, Hilden, Germany). Contaminating DNA was removed using the RNase‐Free DNase set (Qiagen). The expression of two lytic (BZLF1 and glycoprotein 350/220) and six latent (EBNA1, EBNA2, LMP1, LMP2, EBER1, and BARTs) EBV genes was quantified by one‐step multiplex real‐time RT‐PCR using QuantiFast Multiplex RT‐PCR kits (Qiagen) and an Mx3000P instrument (Stratagene, La Jolla, CA) as described previously 36, 37, 38. The relative expression of EBV genes were determined by normalization to *β*2‐microglobulin mRNA.

## Results

### Duvelisib suppresses growth of EBV‐positive and ‐negative B cell lines

**Figure 1:**
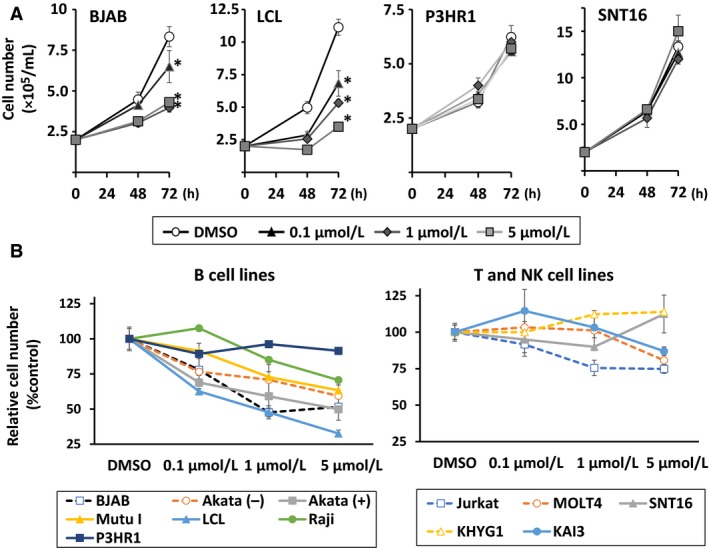
Effects of duvelisib on growth in B, T, and NK cell lines. (A) BJAB, LCL, P3HR1, and SNT16 cells were treated with the indicated concentrations of duvelisib. Viable cells were counted at the indicated times, using the trypan blue exclusion test. Values are expressed as means ± SE of the results from triplicate experiments. *P <* *0.05 as compared with DMSO‐treated cells at 72 h. (B) B, T, and NK cell lines were treated with the indicated concentrations of duvelisib for 72 h. Cell number after treatment is shown as the ratio of the cell number in the different treatment groups to DMSO‐treated cells. Values are expressed as means ± SE of the results from triplicate experiments. NK, natural killer.

To determine the effects of duvelisib on EBV‐negative B cell lines [BJAB and Akata (‐)], EBV‐positive B cell lines [Akata (+), Mutu I, LCL, Raji, and P3HR1], EBV‐negative T cell lines (MOLT4 and Jurkat), EBV‐positive T cell line (SNT16), EBV‐negative NK cell line (KHYG1), and EBV‐positive NK cell line (KAI3), cell lines were treated with 0.1–5 *μ*mol/L of duvelisib and viable cells were counted after 48 and 72 h. We found that duvelisib inhibited cell growth in EBV‐positive and ‐negative B cell lines, except in P3HR1, in a dose‐dependent manner (Fig. 1A and B). Cell growth inhibition of EBV‐negative T cell lines Jurkat, MOLT4, and EBV‐positive NK cell line KAI3, was also observed at 1 or 5 *μ*mol/L of duvelisib. However, no growth inhibition was observed in EBV‐positive SNT16 and EBV‐negative KHYG1. Overall, B cell lines were more susceptible to duvelisib than T and NK cell lines, and further experiments with duvelisib were focused on EBV‐positive and ‐negative B cell lines.

### Effect of idelalisib on EBV‐positive and negative cell lines

**Figure 2:**
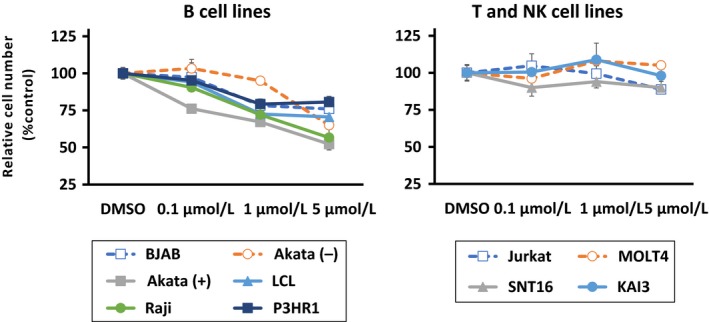
Effects of idelalisib on growth in B, T, and NK cell lines. B, T, and NK cell lines were treated with the indicated concentrations of idelalisib for 72 h. Cell number after treatment is shown as the ratio of the cell number in the different treatment groups to DMSO‐treated cells. Values are expressed as means ± SE of the results from triplicate experiments.NK, natural killer.

To confirm the effects of the selective PI3K*δ* inhibitor idelalisib on B, T, and NK cell lines, 0.1–5 *μ*mol/L of idelalisib was used to treat cells. Viable cells were counted after 72 h of treatment (Fig. 2). Growth of EBV‐positive and –negative B cell lines was inhibited by idelalisib in a dose‐dependent manner. On the other hand, no or modest growth inhibition was observed in T and NK cell lines.

### Duvelisib inhibits activation of the PI3K/Akt signaling pathway in EBV‐positive (+) and –negative (−) B cell lines

**Figure 3:**
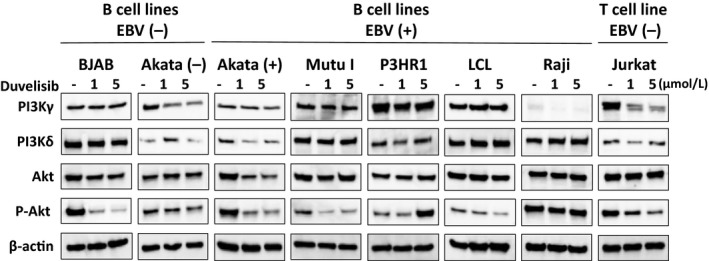
Effects of duvelisib on the PI3K/Akt pathway in B and T cell lines. EBV‐negative B cell lines [BJAB and Akata (‐)], EBV‐positive B cell lines [Akata (+), Mutu I, LCL, Raji, and P3HR1], and EBV‐negative T cell line (Jurkat) were treated without (‐) or with 1 or 5 *μ*mol/L duvelisib for 48 h. Cell lysates were then immunoblotted for the indicated proteins involved in PI3K/Akt signaling.

To confirm activation of the PI3K/Akt signaling pathway, we examined the status of PI3K*γ*, PI3K*δ*, and phospho‐Akt (Thr308) in cell lines (Fig. 3). PI3K*γ* expression was low in Raji cells, but was detected in all other cell lines tested. PI3K*δ* was detected in all the cell lines that were tested. Duvelisib treatment decreased the expression level of PI3K*γ* or PI3K*δ* in Akata (−), Akata (+), and Jurkat. Conversely, the phosphorylated form of Akt was detected in all cell lines tested, indicating activation of Akt regardless of EBV status. Duvelisib treatment induced the inhibition of Akt phosphorylation in five of eight tested cell lines [BJAB, Akata (+), Mutu I, LCL, and Jurkat] (Fig. 3).

### Duvelisib induces apoptosis in EBV‐positive and ‐negative B cell lines

**Figure 4:**
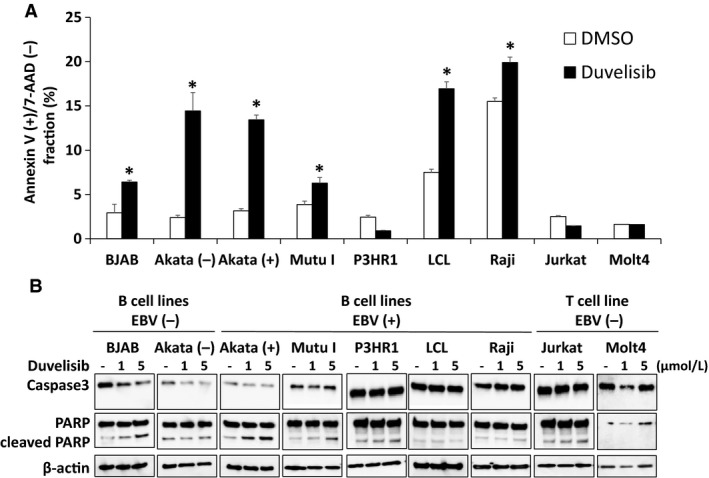
Effects of duvelisib on apoptosis in B and T cell lines. (A) B and T cell lines were treated with DMSO or 5 *μ*mol/L duvelisib for 48 h, and then apoptosis was evaluated by Annexin V/7‐AAD staining using flow cytometry. Values are expressed as means ± SE of the results from triplicate experiments. *P <* *0.05 as compared with DMSO‐treated cells. (B) B and T cell lines were treated with DMSO or 5 *μ*mol/L duvelisib for 48 h, and cell lysates were immunoblotted for caspase‐3 and poly ADP ribose polymerase.

To investigate whether duvelisib induces apoptosis, Annexin V/7‐AAD staining of various duvelisib‐treated cells was evaluated. Duvelisib treatment increased the number of apoptotic cells (Annexin V‐positive and 7‐AAD‐negative), compared to DMSO control treated cells in six of seven tested B cell lines (Fig. 4A). However, duvelisib did not induce apoptosis in B cell line P3HR1 and T cell lines (Jurkat and MOLT4).

The cleavage of caspase‐3 and PARP was also investigated in B and T cell lines to identify apoptosis after duvelisib treatment for 48 h. Duvelisib treatment induced cleavage of PARP in BJAB, Akata (+), and Mutu I (Fig. 4B). Decreased levels of full‐length caspase‐3 were observed in BJAB and Akata (−), whereas cleaved caspase‐3 was not observed in any cell lines. The combined results of flow cytometry and immunoblotting suggest that duvelisib induces apoptosis in EBV‐negative and ‐positive B cell lines, whereas it induces no apoptosis in T cell lines.

### Duvelisib induces G1 cell cycle arrest in B and T cell lines

**Figure 5:**
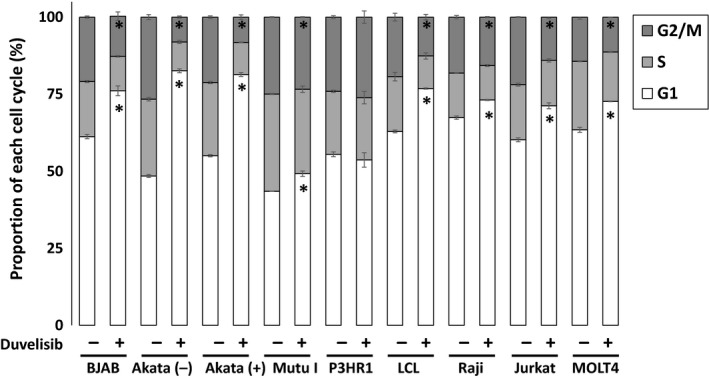
Effects of duvelisib on cell cycle arrest in B and T cell lines. B and T cell lines were treated with DMSO or 5 *μ*mol/L duvelisib for 48 h, and then fixed and stained with propidium iodide. Cell cycle profiles were assessed using flow cytometry. Values are expressed as means ± SE of the results from triplicate experiments. *P <* *0.05 as compared with DMSO‐treated cells.

We next examined the effect of duvelisib on cell cycle distribution in EBV‐positive and ‐negative B cells using flow cytometry analysis with PI staining. Duvelisib treatment induced an increase in cells in the G1 phase and a decrease in cells in the S and G2/M phases in all cell lines tested except P3HR1 (Fig. 5). These results indicate that duvelisib induces G1 cell cycle arrest in B and T cell lines resulting in inhibition of proliferation.

### Duvelisib suppresses the expression of lytic EBV genes in B cell lines

**Figure 6:**
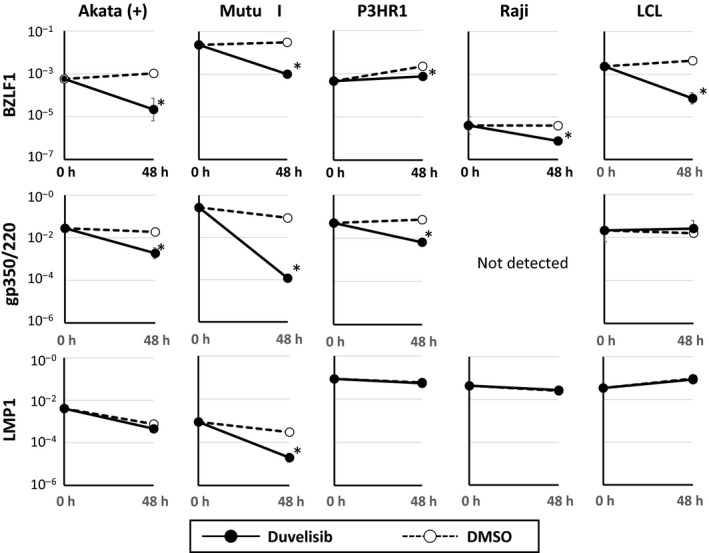
Effects of duvelisib on the expression of EBV‐encoded genes in EBV‐positive B cell lines. EBV‐positive B cell lines [Akata (+), Mutu I, Raji, LCL, and P3HR1] were treated with 5 *μ*mol/L duvelisib and harvested for 48 h to evaluate gene expression using real‐time RT‐PCR. BZLF1 is an immediate early gene and gp350/220 is a late gene in the lytic infection cycle of EBV. LMP1 is a latent EBV gene. *β*2‐Microglobulin was used as an internal control and as a reference gene for relative quantification and was assigned an arbitrary value of 1 (10°). Values are expressed as means ± SE of the results from triplicate experiments. *P <* *0.05 as compared with DMSO‐treated cells.

We next investigated the effect of duvelisib on the expression of eight viral genes in B cell lines. The expression of BZLF1, which is an immediate‐early lytic EBV gene, significantly decreased after duvelisib treatment in all EBV‐positive B cell lines tested (Fig. 6). Furthermore, the expression of the late lytic EBV gene gp350/220 decreased significantly in Akata (+), Mutu I, and P3HR1. In contrast, the expression of LMP1, a latent oncogene of EBV, was decreased in Mutu I. However, this effect was not observed in other cell lines (Fig. 6). Duvelisib treatment did not induce remarkable change in the expression of the other five latent genes (EBNA1, EBNA2, LMP2, EBER1, and BARTs) (data not shown). Taken together, these results suggest that duvelisib suppresses the lytic cycle of EBV, but has little effect on the latent cycle.

## Discussion

In this study, we evaluated the antitumor effects of duvelisib on EBV‐associated lymphoma cell lines and found that B cell lines appeared to be more susceptible to duvelisib than T and NK cell lines regardless of the presence of EBV. It was expected that the presence of EBV might be associated with susceptibility to duvelisib because activation of the PI3K/Akt pathway via LMP1 is considered to be one of the key elements of EBV associated malignancies. Compared to EBV‐negative Akata cell lines, inhibition of Akt phosphorylation by duvelisib was more clearly observed in EBV‐positive cell lines. However, no remarkable differences were observed in susceptibility to duvelisib between EBV‐positive and negative B cell lines. It might be possible that LMP1 or other EBV proteins have only a small effect on PI3K/Akt pathway activation in these cell lines.

LCL, which is classified as type III EBV latency, was the most susceptible to duvelisib among B cell lines tested. However, the association between latency pattern and susceptibility was not clear in this study. Interestingly, P3HR1 was insensitive to duvelisib despite that fact that it harbors an EBNA2‐deficient EBV strain. One study has shown that EBNA2 upregulates Akt activation by inducing miR‐21 expression 39. Although the phosphorylated form of Akt was detected in P3HR1 by immunoblotting, cellular proliferation of R3HR1 might be less dependent on the PI3K/Akt signaling pathway than other EBV‐positive B cell lines.

Most of the B cell lines used in this study were cells originating from Burkitt lymphoma. To our knowledge, this is the first study to show the antitumor effects of duvelisib on Burkitt lymphoma cell lines because antitumor effects of duvelisib have been exclusively investigated in CLL 20, 21, 23. Regarding idelalisib, a selective PI3K*δ* inhibitor, a recent study has shown that antiproliferative effects on EBV‐positive and ‐negative Burkitt lymphoma cell lines (Namalwa and Ramos, respectively) were equivalent to its effects on CLL cell lines 40. While c‐MYC deregulation is a hallmark of Burkitt lymphoma, synergy between constitutive PI3K/Akt signaling pathway activation and c‐MYC has been shown. This suggests that the PI3K/Akt signaling pathway can be a therapeutic target in Burkitt lymphoma 41.

It was expected that duvelisib would have antitumor effects on T or NK cell lines as well as B cell lines because duvelisib is a dual inhibitor of PI3K*γ* and PI3K*δ*. Compared to idelalisib, a selective PI3K*δ* inhibitor, duvelisib showed slightly more cell growth inhibition of T cell lines such as Jurkat or MOLT4. However, cell growth inhibition by duvelisib was modest in T or NK cell lines. Overall, the antitumor effects of idelalisib and duvelisib were similar in the cell lines that were tested. Furthermore, duvelisib did not induce apoptosis in T cell lines. On the other hand, G1 cell cycle arrest was observed in all B and T cell lines tested except P3HR1. Duvelisib treatment could inhibit T cell proliferation by inducing cell cycle arrest. However, its antitumor effects on T cells were limited because apoptosis was not induced.

We found that duvelisib treatment reduced the expression of BZLF1 and gp350/220 mRNA in EBV‐positive B cell lines, suggesting that duvelisib suppresses the lytic cycle of EBV. In EBV‐positive B cell lines, BCR signaling induces BZLF1 activation, and previous studies have shown that PI3K inhibitors such as wortmannin and idelalisib inhibit the EBV lytic cycle 42, 43. Our results are in line with these previous studies, and duvelisib may have specific effects on EBV‐positive B cell lines. In general, the EBV latent cycle is associated with tumorigenesis, and among EBV latent proteins, LMP1 is considered to be a major EBV oncoprotein 5. Induction of the EBV lytic cycle by agents like proteasome inhibitors or histone deacetylase inhibitors could be therapeutically beneficial to EBV‐associated malignancies 44, 45. Furthermore, previous reports have shown that induction of the EBV lytic cycle by chemotherapeutic agents enhanced antiviral nucleoside drug susceptibility to EBV positive tumor cells, suggesting that combination of chemotherapy and antiviral drug may provide a new therapeutic approach for EBV‐associated malignancy 46, 47. Conversely, some reports have shown that the EBV lytic cycle may play a role in tumorigenesis in EBV‐associated malignancies 48, 49. However, it is unclear whether inhibition of the EBV lytic cycle could be a therapeutic target of EBV‐associated malignancies. In this study, suppression of the EBV lytic cycle was observed in P3HR1, which was insusceptible to duvelisib, and EBV‐negative B cell lines showed similar susceptibility to duvelisib. It might be possible that inhibition of the EBV lytic cycle does not play an important role in antitumor effects of duvelisib on EBV‐positive cell lines.

In conclusion, we demonstrated that the PI3K/Akt signaling pathway was activated in EBV‐positive and ‐negative B cell lines. Further, duvelisib treatment induced apoptosis and cell cycle arrest. Although some progress has been achieved with treatments for EBV‐associated lymphomas such as anti‐CD20 antibody and adoptive EBV‐specific cytotoxic T lymphocyte transfer, the effects of these new therapies are still restricted. Our results may indicate that duvelisib has therapeutic potential for the treatment of EBV‐associated B cell lymphomas.

## Conflict of Interest

None declared.
